# Hostile: accurate decontamination of microbial host sequences

**DOI:** 10.1093/bioinformatics/btad728

**Published:** 2023-12-01

**Authors:** Bede Constantinides, Martin Hunt, Derrick W Crook

**Affiliations:** NDM Experimental Medicine, University of Oxford, John Radcliffe Hospital, Oxfordshire OX3 9DU, United Kingdom; The National Institute for Health Research Health Protection Research Unit in Healthcare Associated Infections and Antimicrobial Resistance, University of Oxford, John Radcliffe Hospital, Oxfordshire OX3 9DU, United Kingdom; NDM Experimental Medicine, University of Oxford, John Radcliffe Hospital, Oxfordshire OX3 9DU, United Kingdom; EMBL-EBI, Wellcome Genome Campus, Cambridgeshire CB10 1SD, United Kingdom; NDM Experimental Medicine, University of Oxford, John Radcliffe Hospital, Oxfordshire OX3 9DU, United Kingdom; The National Institute for Health Research Health Protection Research Unit in Healthcare Associated Infections and Antimicrobial Resistance, University of Oxford, John Radcliffe Hospital, Oxfordshire OX3 9DU, United Kingdom; The National Institute for Health Research Oxford Biomedical Research Centre, University of Oxford, John Radcliffe Hospital, Oxfordshire OX3 9DU, United Kingdom

## Abstract

**Motivation:**

Microbial sequences generated from clinical samples are often contaminated with human host sequences that must be removed for ethical and legal reasons. Care must be taken to excise host sequences without inadvertently removing target microbial sequences to the detriment of downstream analyses such as variant calling and *de novo* assembly.

**Results:**

To facilitate accurate host decontamination of both short and long sequencing reads, we developed Hostile, a tool capable of accurate host read removal using a laptop. We demonstrate that our approach removes at least 99.6% of real human reads and retains at least 99.989% of simulated bacterial reads. Using Hostile with a masked reference genome further increases bacterial read retention (≥99.997%) with negligible (≤0.001%) reduction in human read removal performance. Compared with an existing tool, Hostile removes 21%–23% more human short reads and 21–43 times fewer bacterial reads, typically in less time.

**Availability and implementation:**

Hostile is implemented as an MIT-licensed Python package available from https://github.com/bede/hostile together with supplementary material.

## 1 Introduction

Microbial specimens are often contaminated with host sequences. Since experimental host genome depletion protocols are imperfect, host DNA often reaches the sequencing instrument. Where the specimen host is a human, it is important that host sequences are subsequently deleted in order to protect anonymity. The widespread human contamination of publicly deposited microbial sequence data (Bush *et al.* 2020) is therefore regrettable and raises regulatory concerns, particularly in light of the rapid growth of metagenomic diagnostics. Furthermore, unwanted host sequences waste computing resources and may adversely affect downstream analyses such as variant calling and *de novo* assembly. Host decontamination is therefore the first step performed in many microbial genomic analyses. Existing approaches employ one of three strategies: (i) exclusive retention of reads aligning to a target microbial genome (Hunt *et al.* 2022), (ii) subtractive removal of reads aligning to a host genome, and (iii) subtractive removal after metagenomic read classification (Kim *et al.* 2016, Wood *et al.* 2019). Where the target microbe is known *a priori*, the first strategy (exclusive retention) may be most suitable: for SARS-CoV-2 it is both more accurate and computationally efficient than subtractive removal (Hunt *et al.* 2022). However, the second and third strategies (subtractive removal) are generalisable, and thus necessary for analysis of microbes that are unknown *a priori*, mixtures, or novel.

In this article, we describe a simple tool implementing subtractive removal of contaminant human genome sequences, together with rigorous evaluation of its performance against real human genomes from the 1000 Genomes Project and simulated bacterial reads representing the 985 complete bacterial assemblies in the FDA-ARGOS dataset (Sichtig *et al.* 2019). We also report performance using simulated reads for 140 complete mycobacterial genomes. These results provide evidence of the accuracy of the approach in terms of both its ability to remove human host reads (sensitivity), and to retain microbial reads (specificity).

## 2 Materials and methods

Hostile is implemented as a Python package providing a command line interface and Python API. The decontamination process involves a series of streaming operations on optionally gzip-compressed input FASTQ files: (i) alignment to a custom human reference genome (Minimap2 or Bowtie2), (ii) counting distinct reads (Samtools), (iii) discarding aligned reads (and their mate reads for paired data; Samtools), (iv) counting remaining reads (Samtools), (v) Optionally replacing read names with incrementing integers (Awk), and (vi) writing gzip-compressed FASTQ files (Samtools) (Langmead and Salzberg 2012, Li 2018, Danecek *et al.* 2021). These operations are streaming so as to minimize reading from and writing to storage, reducing overall execution time. Alignment of each read ceases after a single high-quality match to the reference genome is found. For Bowtie2, default alignment parameters are used, while for Minimap2 a minimum chaining score of 40 is enforced for both long and short reads. Bowtie2 (GPL-licensed) is the default aligner for short reads due to its relatively compact (<4 GB) random access memory (RAM) footprint, while Minimap2 (MIT-licensed) is the default aligner for long reads, requiring approximately 13 GB of RAM when using the map-ont preset for ONT (Oxford Nanopore Technologies) reads. Hostile generates summary statistics in JSON format including the total number of reads before and after decontamination. For ease of installation, Hostile is available as a Docker container and is packaged with Bioconda.

A custom human reference genome was built from the T2T-CHM13v2.0 human genome assembly (Nurk *et al.* 2022) and human leukocyte antigen (HLA) sequences. Human Illumina 2x100bp (Eberle *et al.* 2017) and ONT (Jain *et al.* 2018) reads from the well-characterised NA12878 sample were downsampled using BBTools (Bushnell 2014) to a target depth of 10. We also examined 26 Illumina 2x150bp genomes at 30x depth representing each of the populations in the expanded 1000 Genomes Project, originating from Africa, Asia, Europe, and the Americas (Byrska-Bishop *et al.* 2022), in addition to newer data for the NA12878 sample. For FDA-ARGOS bacterial (*n *=* *985) and mycobacterial (*n *=* *140) metagenomes, Illumina reads were simulated with DWGSIM (Homer 2010) while ONT reads were simulated with PBSIM2 (Ono *et al.* 2021). Refer to the Supplementary Text for detailed information about test data, masked reference genome construction, and read simulation.

We evaluated Hostile version 0.0.2 performance with default and masked (human-t2t-hla-argos985-mycob140) databases, alongside the NCBI Human Read Removal Tool (HRRT) version 2.1.0 with its default database (https://github.com/ncbi/sra-human-scrubber). Testing was performed using a virtual machine running Ubuntu Linux 22.04 with 128 GB of RAM and an AMD EPYC (x86-64 architecture) processor. In order to address a defect in HRRT’s handling of paired reads and restore intended behaviour, BBTools was used to remove singleton reads from HRRT output.

## 3 Results

Full benchmark results are shown in Supplementary Tables S1 and S2, summarised in Table 1, and described here. Table 1 includes results for Hostile run with a masked database. Supplementary Table S3 includes accession numbers for all datasets used. Refer to the Supplementary Text for detailed information about test data preparation.

**Table 1.** Evaluation of Hostile and the NCBI Human Read Removal Tool (HRRT) on real human and simulated bacterial and mycobacterial reads.^a^

Dataset	Samples	Total reads	Reads retained (%)		Execution time (s)		Peak RAM (GB)	
			Hostile	HRRT	Hostile	HRRT	Hostile	HRRT
Human Illumina 10x (real)	1	404 580 418	0.1320%	0.1595%	1378	901	4	18
Human ONT 10x (real)	1	2 498 111	0.0381%	0.0373%	2740	414	14	1
Human Illumina 30x (real)	27	20 772 464 024	0.3936%	0.5331%	401 895	100 421	4	71
Bacteria Illumina	985	273 511 602	99.9999%	99.9975%	1314	4491	4	1
Bacteria ONT	985	8 230 970	99.9970%	99.9890%	1855	4617	13	1
Mycobacteria Illumina	140	51 360 128	100.0000%	99.9998%	230	785	3	1
Mycobacteria ONT	140	1 544 982	100.0000%	99.9986%	394	773	13	1

a Percentages of retained reads represent the sum of reads from all samples in the dataset.

**Accuracy of human read removal:** For 10x depth 2x100bp Illumina data for NA12878, Hostile retained 0.132% of human reads while HRRT retained 0.160% (21% more). For 30x depth 2x150bp Illumina data for 27 representative genomes of each population in the expanded 1000 Genomes Project (plus NA12878), Hostile retained 0.594% of human reads overall, while HRRT retained 0.729%, a mean increase of 23% (95% CI: 16%, 31%; see Supplementary Table S4 for individual figures). Surprisingly, human retention for NA12878 in these 30x 2x150bp data was considerably higher than in the older 10x 2x100bp data (0.132% versus 0.641%). To investigate this discrepancy we performed *de novo* assembly (Li *et al.* 2015), revealing Epstein-Barr Virus (EBV) representation in all of the 27 studied Byrska-Bishop *et al.* genomes. Subtraction of EBV reads using Hostile with a custom index comprising accessions NC_007605.1 and NC_009334.1 enabled generation of adjusted accuracy figures, and highlighted one accession (ERR3242202) with 1.3% EBV contamination. Following EBV subtraction, retention for 2x150bp NA12878 decreased from 0.641% to 0.406% for Hostile and from 0.689% to 0.458% for HRRT, with overall human retention for the 27 2x150bp genomes decreasing from 0.594% to 0.393% for Hostile. For ONT data, Hostile retained 0.038% of reads while HRRT retained 0.037% (2% more). Using Hostile with a reference genome masked against bacterial sequences marginally increased retention of human reads from 0.131738% to 0.131979% (Illumina 2x100bp) and from 0.038029% to 0.038069% (ONT).

**Accuracy of microbial read retention:** Accuracy of bacterial read retention was evaluated using Illumina and ONT sequences simulated from reference-grade complete bacterial assemblies in the FDA-ARGOS dataset. For simulated Illumina data, Hostile retained 99.99989% of reads while HRRT retained 99.99752%, corresponding to HRRT removing a mean of 21 times (95% CI: 9, 30) more bacterial reads than Hostile (see Supplementary Table S4 for detailed figures). Hostile’s bacterial read retention was further increased to 99.99994% through use of a reference genome masked against bacterial sequences. For simulated ONT data, Hostile and HRRT retained similar percentages of bacterial reads—99.98918% and 99.98901% respectively. Use of a masked reference genome further reduced the number of bacterial ONT reads removed by Hostile from 891 to 251 (43 times less than HRRT). For mycobacterial reads, 140 complete assemblies were simulated in the same fashion. For simulated mycobacterial Illumina data, Hostile retained 99.99998% of reads while HRRT retained 99.999751%, corresponding to HRRT removing 15 times more mycobacterial reads than Hostile. For simulated mycobacterial ONT data, Hostile retained 99.99948% of reads while HRRT retained 99.99864%. Use of a masked reference with Hostile resulted in perfect (100%) retention of both Illumina and ONT reads.

**Execution time and memory usage:** Execution time was measured as the wall clock time required to process gzip-compressed FASTQ input and create gzip-compressed decontaminated FASTQ output with eight threads. Median execution times and peak memory usage figures are shown in Table 1, and full results for individuals’ trials can be found in Supplementary Table S1. Three trials were performed for datasets other than the 27 2x150bp human Illumina genomes. For decontaminating simulated bacterial Illumina reads, Hostile was faster, with HRRT taking 242% (95% CI: 219%, 266%) longer than Hostile. Hostile was also faster at decontaminating simulated bacterial ONT reads, with HRRT taking 153% (95% CI: 120%, 187%) longer than Hostile. This was also the case with simulated mycobacterial reads, with HRRT taking 241% (95% CI: 218%, 264%) and 96% (95% CI: 89%, 104%) longer than Hostile to decontaminate Illumina and ONT reads respectively. For decontaminating real human Illumina reads, HRRT was faster than Hostile, with Hostile taking 55% (95% CI: 51%, 58%) longer than HRRT. HRRT was also faster at decontaminating human ONT reads, with Hostile taking 564% (95% CI: 553%, 574%) longer than HRRT. Decontaminating the 27 2x150bp human Illumina genomes with HRRT took 28h versus 112h for Hostile. Hostile had stable memory requirements, using 3–4 GB of RAM when processing short reads (Bowtie2) and 13–14 GB when processing long reads (Minimap2). In contrast, HRRT’s memory usage varied between 1 and 71 GB depending on the quantity of human host reads present in the dataset, necessitating use of a virtual machine instance with 128 GB of RAM in order to individually process the 27 2x150bp genomes from the 1000 Genomes Project. Unlike Hostile, HRRT generated uncompressed intermediate versions of all input FASTQ files during operation, temporarily using considerable additional disk storage.

## 4 Discussion

In any diagnostic or experiment where microbial genomes might be contaminated with human genomes, host decontamination is necessary both to safeguard patient anonymity and to avoid encumbering downstream analyses with redundant and potentially detrimental off-target sequences. For downstream analysis it is also of critical importance that microbial sequences are not inadvertently removed, leading to false negative variant calls and incomplete *de novo* assemblies. Where target microbes are unknown *a priori*, mixed or sufficiently novel, a subtractive human read removal approach is required, involving non-trivial computation using gigabytes of RAM. Hostile uses one of two complementary seed-and-extend aligners to accurately excise human reads. Bowtie2 is well-suited for decontaminating short reads due to its small memory footprint, fast index loading, and memory-mapped index support, while Minimap2 offers excellent long (and short) read performance in return for a larger index that is considerably slower to load. Both implementations take advantage of multiple processor cores, enabling Hostile to perform decontamination of host-light reads faster than HRRT. Compared with HRRT, Hostile is more sensitive in terms of removing human reads, and produces an order of magnitude fewer false positives, effectively retaining diverse bacterial reads, even without the use of a masked reference genome. Masked reference genomes further reduce false positive rate, and can be easily created using a built-in utility, with prebuilt masked references available to download.

While currently more accurate base-for-base, short reads present a greater challenge for decontamination due to their relatively low information content. Nevertheless, for a catalogue of short read genomes representing diverse human populations, Hostile removed 99.6% of reads. Although this figure accounts for widespread Epstein-Barr Virus contamination (>1% in ERR3242202), other non-human DNA likely accounts for a significant proportion of the remaining 0.4%. The figure of 99.6% should therefore be considered a lower bound for sensitivity with short reads.

Unlike existing tools, Hostile streams compressed FASTQ input to compressed FASTQ output without creating intermediate files. Hostile’s RAM requirements are increasingly met by consumer laptops, creating scope for accurate client-side host decontamination using what we hope will be broadly useful software.

## Supplementary Material

btad728_Supplementary_Data

## Data Availability

Source code is available from https://github.com/bede/hostile and archived at https://zenodo.org/record/8169826. International Nucleotide Sequence Database Collaboration (INSDC) accession numbers for sequencing datasets used in this article are provided in the permanently archived online supplementary material.
